# Physicochemical and microbiological changes during two-stage fermentation production of umqombothi

**DOI:** 10.1016/j.heliyon.2024.e24522

**Published:** 2024-01-13

**Authors:** Thembelani Xolo, Zanephyn Keyser, Victoria A Jideani

**Affiliations:** Department of Food Science and Technology, Cape University of Technology, Bellville Campus (Main), Symphony Road, 7530, South Africa

**Keywords:** Umqombothi, Fermentation, Traditional beverage, Lactic acid bacteria, Alcoholic beverage

## Abstract

Umqombothi is a traditional South African fermented beverage. The brewing process limits its consumption to a day or two after production due to the constant production of carbon dioxide. In this study the physicochemical and microbial changes in Umqombothi produced at two-stage fermentation temperatures [U1 (30-30 °C), U2 (30-25 °C), U3 (25–30 °C)] were studied over 52 h. Samples were collected before first fermentation (BFF), after first fermentation (AFF), before second fermentation (BSF), after second fermentation (ASF) and after final product (FP). For all three fermentation temperatures, there was a significant increase (*p* < 0.05) in microbial counts and a significant drop in pH following fermentation stages (AFF and ASF), with a considerable decrease in total soluble solids (TSS) after ASF. The total viable count (TVC), lactic acid bacteria (LAB), yeast, and mould were not detected in the BSF samples for all three fermentation temperatures. The LAB count was significantly (*p* < 0.05) different at 5.18, 5.36 and 5.25 log CFU/mL for U1, U2 and U3, respectively. The pH was 3.96, 4.12 and 4.34 for U1, U2 and U3, respectively, and was significantly (*p* < 0.05) different. Total soluble solids significantly (*p* < 0.05) increased at the BSF at all temperatures. There was no significant (p > 0.05) difference in specific gravity and ethanol content of Umqombothi at all fermentation temperatures. At all fermentation temperatures, Umqombothi was characterised by redness and yellowness, with that collected from U1 being the lightest in colour (L* = 71.24). Colour difference (ΔE) in the between of 4–8 was perceivable but acceptable as they had a ΔE value of 3.58, 2.07 and 2.02 for U1–U2, U1–U3 and U2–U3 respectively. Umqombothi produced at 30 °C for first and second fermentation (U1) was the most preferred by the consumer panellist and consequently, the best fermentation temperature to produce Umqombothi.

## 1 Introduction

Hundreds of years before handcrafted artisan beverages became fashionable, Africans brewed homemade beverages from locally available ingredients. Indigenous fermentation methods in Africa are, at best, an art form, with quality varying according to the kind and quality of raw materials employed, ambient conditions, and containers used [[1], [2], [3], [4]]. Umqombothi remains one of the oldest and most popular traditional beverages among black South Africans. The ingredients are locally available, and it is produced by local farmers. Umqombothi also plays a fundamental role in traditional brewing history, people's culture and beliefs [[5], [6], [7]].

Umqombothi is created with a mixture of water, yeast, maize malt and sorghum malt (red). The ingredients are combined in a stainless steel bucket; the resulting drink has a unique pleasant sour yoghurt flavour and is significantly thicker than clear beer [8].

Umqombothi is a B vitamin-rich beverage [2,5,9,10] brewed mostly by women either for marketing purposes, or to be consumed at social gatherings, weddings, or ritual events [11,12]. The colour varies from pale buff to pink-brown to cream-colour after sieving and has a flavour similar to yoghurt [8]. It has a short shelf life of 2–3 days, alcoholic content of 2–3% [2,8]*,* pH of 3.3–4.2, and 0.02–0.26 % lactic acid content. The beverage should be consumed in its active state of fermentation since the composition constantly changes due to the frequent production of carbon dioxide [6,10], while the development of acetic acid results in an acidic and poor flavour. Lactic acid bacteria (LAB) and yeast are the predominant microorganisms involved during fermentation [1,13]. Lactic acid bacteria are mainly responsible for souring the product, while yeast is necessary for alcohol production. The art of brewing lies within the challenge to produce enough nutrients for LAB and yeast to proliferate, but too much degradation of the starch is not allowed, as it could cause the beverage to be thin. The production process involves fermentation or souring, boiling, followed by a second fermentation under ambient conditions [10,14]. The beverage is commonly unstable, and thus its organoleptic traits fluctuate. In addition, the traditional Umqombothi fermentation process is carried out under uncontrolled conditions (e.g., uncontrolled temperature and a non-sterile environment). The challenge lies in consistently producing high-quality beer with a long shelf life [5,14].

Umqombothi's production process has not changed from its usual fermentation at ambient temperatures, cooking at unstandardized temperatures and time, and pitching a back slope culture containing unknown bacterial strains, from a previous overnight brew. The whole process is dependent on the brewer's judgment [10,14]. The production process mainly lacks controlled hygienic practices as well as a standardised method. The effective determination of chemical and microbiological content and nutritional composition by Ref. [15] encourages the need to investigate the production of Umqombothi (Xhosa version) under different controlled fermentation temperatures. The objective of this study was to determine the optimal fermentation temperatures of Umqombothi and to evaluate the physicochemical, microbiological, and sensory characteristics of the final product.

### 1.1 Materials and methods

#### 1.1.1 Source of material and equipment

Maize meal, sorghum malt, maize malt, and Mnanti beer powder were obtained from a local supermarket in Bellville, South Africa. Maize malt (Umthombo wombona) was purchased from Boxer Supermarket, Eastern Cape Province (Mdatsane), South Africa. Plate Count Agar (“PCA”, NCM0010A, Neogen culture media, Lasec South Africa), De Man Rogosa and Sharpe (“MRS”, NCM0190A, Neogen culture media, Lasec South Africa), Violet Red Bile Agar ("VRBA", bio lab, Merck South Africa) and Rose-Bengal Chloramphenicol Agar ("RBC", NCM0135A, Neogen culture media, Lasec) were obtained from Merck (Cape Town, South Africa). All brewing equipment used in Umqombothi production was acquired from the Department of Food Science and Technology, Cape Peninsula University of Technology (CPUT), South Africa.

#### 1.1.2 Umqombothi production

**Fig. 1:**
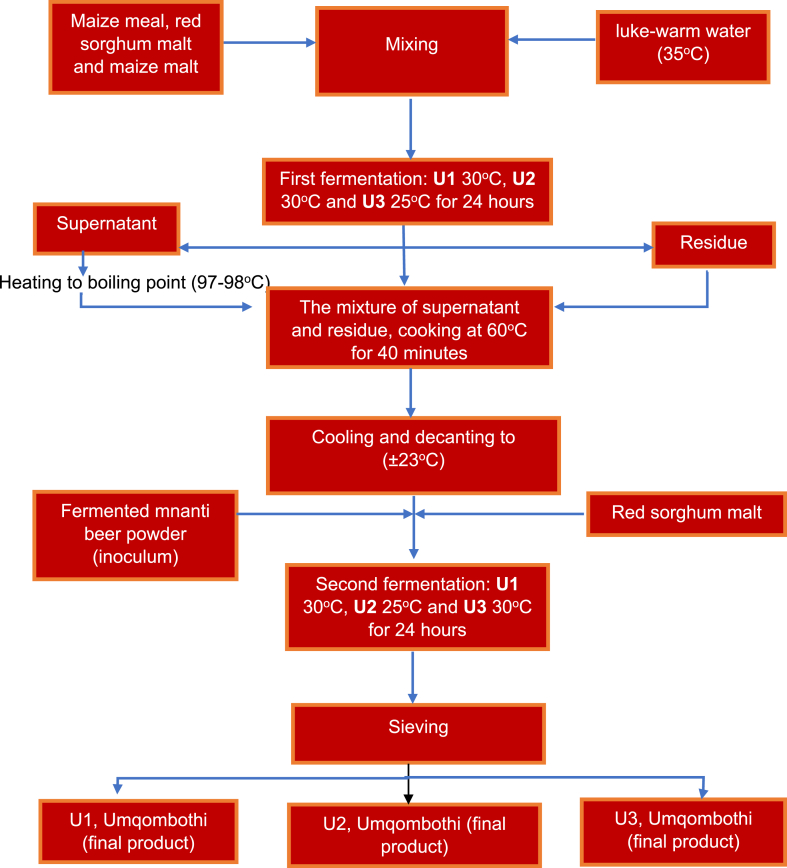
Detailed flow diagram of the Umqombothi production process.

The umqombothi production process is presented in Fig. 1. Three batches of Umqombothi were produced by weighing out 500 g of maize meal, 100 g of red sorghum and 100 g of maize malt in three separate containers labelled U1, U2 and U3. Luke-warm warm water (35 °C) (5 L) was added to each container and mixed to form a homogenous mixture. The three batches were incubated for 24 h, with U1 at 30 °C, U2 at 30 °C and U3 at 25 °C for the first fermentation to occur naturally. After 24 h, the three batches were individually separated into two phases, namely the supernatant and the residue. The supernatant (liquid) of the respective batches was decanted into separate stainless-steel pots and heated to boil and residue (solid particles) from each batch were added to the boiling liquid. The temperature was then reduced to 60 °C while stirring for 40 min until a porridge was formed. The porridge was cooled down to room temperature, and 300 g of red sorghum malt and 75 g of beer powder in 250 mL water were used as an inoculum (the mixture of beer powder and water was incubated at 25 °C for 24 h before being added). The three batches were incubated for 24 h, with U1 at 30 °C, U2 at 25 °C and U3 at 30 °C. This incubation was referred to as the second fermentation. Thereafter, the products were removed from the incubation chamber and strained through a sieve with a pore size of ca 0.55 mm. The supernatant of each batch, referred to as Umqombothi, was transferred to a labelled sterile container while the residue was discarded.

Triplicate samples for each batch were collected before and after the first fermentation, after cooking, after the second fermentation and after the sieving process. The samples were collected using a sterile sampling cup and stored at refrigeration temperature (4–6 °C) before analysis. A sensory evaluation was conducted on the final product samples for each batch. The samples were evaluated for physicochemical, chemical, microbiological and sensory analysis [16].

### 1.2 Physicochemical analysis

#### 1.2.1 Colour

The colour of Umqombothi was evaluated using a Konica Minolta Spectrometer CM – 5 [Norich (pty) Ltd (Japan)], serial no:1101313, 45°/0° standards, set at standard observer 10° and D65. The instrument was zero calibrated using black (L* = 5.49, a* = 7.08, b*4.66) and white (L* = 93.41, a* = 1.18, b* = 0.75) tiles. Umqombothi 3 ml/3 g was deposited in a light-coloured sample holder, and the reflections were measured on the L*a*b* and LCh colour scales. The L* coordinate is lightness, where a value of 100 represents whiteness, and 0 is a representation of blackness. Coordinate a* referred to the green(−)/red(+) chromatic and coordinate b* referred to the blue(−)/yellow(+) chromatic. Measurements for each sample were performed in triplicate. C* (Chroma) and h (hue) angle 0° = +a*, 90 = +b*, 180 = -a* and 270° = -b*) was determined as described by Ref. [17].

#### 1.2.2 pH

The pH for each sample was measured at ambient temperature (23 ± 2 °C) using a calibrated pH meter from Mettler-Toledo GmbH Switzerland (FiveEasy F20). Serial no: B918615210. The glass electrode was calibrated with buffers 7 and 4 (Merck) [18].

#### 1.2.3 Total soluble solids (TSS) and specific gravity

Total soluble solids were measured with a refractometer (Bellingham & Stanley, serial no 036906, UK, 0–50 % °Brix). Specific gravity was measured using a Brew craft refractometer [Portable °Brix & Beer wort specific gravity refractometer, handheld (°Brix 0–32, and Gravity 1.000–1.130)] [18]*.* Several drops of the sample were placed on the prism surface. The liquid on the prism must be free of bubbles or floating particles of pulp or other matter. The prism was then closed. To get a valid reading, the instrument turned towards the light. If necessary, the eyepiece was focused until a clear image appears. The position at which the demarcation line and dark regions cross the vertical scale is the value of the percentage of total soluble solids reading.

#### 1.2.4 Ethanol

Ten millilitres of each sample were centrifuged at 11000 rpm for 10 min in an Avanti J-E Centrifuge (Beckman culture, USA), Serial no: JSE11B30 and cat no: 369003. The supernatants acquired from each sample were used for alcohol content determination by gas chromatography (GC) according to the methods of [8] (Gas Chromatograph system 7890A, Agilent Technologies, made in China, serial no CN90352528, G 2614A).

### 1.3 Microbiological analysis

Enumeration of the total viable count (TVC), LAB, Coliforms, yeast, and moulds in Umqombothi were performed according to the methods described by SANAS 4833:2007, ISO: 4833:2007. The pour plate method was employed for the enumeration of microorganisms in Umqombothi. Counting of all typical colonies was performed using a ColonyStar colony counter from Funke GERBER labortechnik (Berlin, Germany), serial no:8500-6107 and control (Positive controls for VRBA, MRS, RBC, and SPCA were performed by streaking with *Escherichia. coli*, *Lactobacillus gasseri*, and yeast (*Saccharomyces cerevisiae*), mould (*Aspergillus* spp). Controls for (the ringer, stomacher bags and pipette tips) were carried out with 1 ml of ringer into a Petri dish plate (ringers' control), stomacher bag (control), and pipette tips (control) and poured with PCA and incubated at 37 °C for 24 h. Experiments were performed in triplicates. Only plates containing colonies from 30 to 300 were counted [19].

#### 1.3.1 Enumeration of bacteria

Umqombothi (10 g) was weighed into 90 mL sterile Ringer's solution to obtain a 10^−1^ dilution and mixed well. A series of dilutions (10^−1^ to 10^−6^) were subsequently prepared by transferring 1 mL of a dilution into 9 mL sterile Ringer's solution. For each dilution 1 mL aliquot was thereafter aseptically transferred to a sterile Petri dish. Approximately 12–15 mL of pre-cooled plate count agar (PCA), de Man Rogosa and Sharpe Agar (MRS) and violet-red bile agar (VRBA) was poured into the respective Petri dishes and carefully swirled to mix well, for the enumeration of total viable bacteria, total lactic acid bacteria and coliforms. Once all plates were allowed to solidify, they were incubated in an inverted position at 37 °C for 48 hourrs [8].

#### 1.3.2 Enumeration of yeast and moulds

From the series of dilutions in 1 mL aliquot was thereafter aseptically transferred to a pre-labelled sterile Petri dish and approximately 12–15 mL of pre-cooled Rose Bengal chloramphenicol agar (RBC) was poured into each Petri dish plate and swirled to mix well. Once solidified, plates were incubated at 25 °C for 5 days in an inverted position [8].

### 1.4 Sensory evaluation

Sensory evaluation was conducted based on the IFST Guidelines for Ethical and Professional Practices for the Sensory Analysis of Foods. To reduce risks to participants, Umqombothi was assessed for potential microbial, chemical, and physical hazards and certified safe before conducting the sensory evaluation. Ingredients and manufacturing process are similar to what commercial Umqombothi is made. Research activities were restricted to those detailed in the research proposal. Test participants were volunteers, and their right to withdraw from the research was explained to them; informed consent was obtained from research participants, the product ingredient and alcohol content was explained to participants and their anonymity and confidentiality was protected. Umqombothi is a traditional South African alcoholic beverage with an alcohol concentration of 2–3 %. Because of the dangers of drinking to one's health and that it impairs one's ability to drive, panellists were limited to one round of tasting, which were consisted of 90 mL of Umqombothi or around 1.8 ml of alcohol per individual. Panellists were also told that they are not required to finish the product because the goal was to taste rather than drink.

A consumer sensory analysis was performed on the three batches of Umqombothi using 50 untrained panellists in the sensory laboratory of the Food Science and Technology Department at CPUT. The samples were presented in 50 mL white polystyrene cups placed side by side on a plastic tray. Each sample cup contained 30 mL of the respective final product batches and was identified by a three-digit code and served at room temperature (23 ± 2 °C). A cup of water was provided to clear the pallet before and during tastings. The panellists were provided with a score sheet that consisted of three coded samples with a 5-point hedonic scale ranging from 1 = dislike extremely to 5 = like extremely. The panellists were instructed to rate each sample individually on its merit on the five-point hedonic rating scale for appearance, colour, taste, aroma, texture, and overall acceptability [18].

## 2 Data analysis

Multivariate analysis of variance (MANOVA) was used to determine the significant differences (p ≤ 0.05) in attributes among samples. Duncan's multiple range test was used to separate means where a significant difference existed (IBM SPSS version 22, 2021).

### 2.1 Results and discussion

#### 2.1.1 Physicochemical changes in umqombothi

There were five different sampling stages during Umqombothi production. Before first fermentation (BFF), after first fermentation (AFF), before second fermentation (BSF), after second fermentation (ASF) and the final product (FP) (Umqombothi).

The lightness of Umqombothi from different fermentation temperatures, at different sampling stages, is displayed in Table 1. The lightness of sampling stages BFF, AFF, BSF, ASF and FP ranged from 57.11 to 90.60, 54.51–89.12 and 55.17–91.05 for U1, U2 and U3 respectively. The lightness was significantly (*p* < 0.05) different during all sampling stages of the Umqombothi production process for U1, U2 and U3 respectively. The lightness increased considerably (*p <* 0.05), between ASF and FP for U1, U2 and U3, respectively. This could be due to the increase of microbial counts during the respective fermentation stages, elevated temperatures during cooking and the removal of solid particles in the final product, increasing lightness as they make the product lighter. Cooking and fermentation are methods for improving the overall safety of the beverage [10].

**Table 1:** The effect of two-stage fermentation on lightness values of Umqombothi.

sampling stage	Lightness		
	U1 (30-30 °C)	U2 (30-25 °C)	U3 (25–30 °C)
**Before first fermentation (BFF)**	90.60 ± 0.01^a^	89.12 ± 0.02^a^	91.05 ± 0.47^a^
**After first fermentation (AFF)**	87.02 ± 0.00^b^	85.02 ± 0.02^b^	88.80 ± 0.01^b^
**Before second fermentation (BSF)**	58.89 ± 0.01^c^	54.78 ± 0.02^c^	55.17 ± 0.02^c^
**After second fermentation (ASF)**	57.11 ± 0.02^d^	54.51 ± 0.00^d^	55.20 ± 0.00^c^
**The final product (FP)**	62.59 ± 0.36^e^	59.66 ± 0.00^e^	61.68 ± 0.00^d^
**Overall fermentation temperatures**	71.24 ± 15.01^a^	68.62 ± 15.77^b^	70.38 ± 16.72^c^

Mean value ± standard deviation of triplicate determination. Mean values in the same column followed by different letters are significantly (*p* ≤ 0.05) different.

The colour of the wort changes when maize malt and red sorghum malts are introduced as adjuncts in brewing [20]. The values of lightness as affected by fermentation temperatures were 71.24, 68.62, and 70.38 and they were significantly (*p* < 0.05) different for U1, U2 and U3 respectively. Umqombothi U1 had a significantly (*p* < 62.59) greater lightness than U3 and U2. However, higher temperatures for both fermentation processes exhibited a lightness value of 71.24, show that Umqombothi is light in colour, as all the values are close to 70, which is closer to 100 representing the whiteness of the colour scale.

The redness of Umqombothi obtained from different fermentation temperatures, at different sampling stages is displayed in Table 2. According to Ref. [21] the sorghum malt kernel is initially reddish, so this was expected. The redness of sampling stages BFF, AFF, BSF, ASF and FP ranged from 0.93 to 8.79, 1.07–7.73 and 0.98–7.97 for U1, U2 and U3 respectively. The level of a* was significantly (*p* < 0.05) different during all sampling stages of the production process for U1, U2 and U3, respectively. According to Ref. [22] temperature, polyphenol oxidation, and grist material all affect the colour of wort during the processing phases. This could be due to the increase of microbial load during the fermentation stages as well as the effect of heat during cooking. The removal of solid particles significantly (*p* < 0.05) reduced the redness in the final product of all three different fermentation temperatures. This means the product showed more redness than greenness (redness +a*) in the analysis.

**Table 2:** The effect of two-stage fermentation on redness values of Umqombothi.

Sampling stage	Redness		
	U1 (30-30 °C)	U2 (30-25 °C)	U3 (25–30 °C)
**Before first fermentation (BFF)**	0.93 ± 0.01^a^	1.07 ± 0.02^a^	1.03 ± 0.02^a^
**After first fermentation (AFF)**	1.08 ± 0.00^b^	1.24 ± 0.01^b^	0.98 ± 0.01^b^
**Before second fermentation (BSF)**	2.26 ± 0.01^c^	2.15 ± 0.05^c^	1.98 ± 0.01^c^
**After second fermentation (ASF)**	8.79 ± 0.01^d^	7.73 ± 0.00^d^	7.97 ± 0.01^d^
**The final product (FP)**	6.99 ± 0.01^e^	6.30 ± 0.00^e^	6.11 ± 0.00^e^
**Overall fermentation temperatures**	4.01 ± 3.37^a^	3.69 ± 2.87^b^	3.61 ± 2.98^c^

Mean value ± standard deviation of triplicate determination. Mean values in the same column followed by different letters are significantly (*p* ≤ 0.05) different.

The raw material, cooking, and inclusion of sorghum malt and as inoculum after first fermentation could explain the considerable significant (*p* < 0.05) increase of redness during the after-fermentation stages. The sieving (removal of solid particles) significantly (*p* < 0.05) decreased the redness in the FP sampling stage for U1, U2 and U3. The redness as affected by fermentation temperatures was 4.01, 3.69, 3.61 and they were significantly (*p* < 0.05) different for U1, U2 and U3 respectively. The U1 Umqombothi batch had a significantly (*p* < 4.01) greater redness than U2 and U3 respectively.

The yellowness of Umqombothi from different fermentation temperatures, at different sampling stages is displayed in Table 3. The yellowness values of Umqombothi were positive, which suggested that Umqombothi is in the yellowness colour space. This is expected because yellow maize is mostly used in traditional beverages [8,21]. The yellowness of sampling stages of BFF, AFF, BSF, ASF and FP ranges from 4.27 to 23.15, 4.77–22.35 and 4.77–23.30 for U1, U2 and U3, respectively. The yellowness was significantly different during all sampling stages of the Umqombothi production process for U1, U2 and U3, respectively. The yellowness significantly (*p* < 0.05) increased during the sampling stages of U1, BFF (4.27), AFF (5.12), BSF (8.70), ASF (22.73) and FP (23.15) respectively. The significant (*p* < 0.05) increase in microbial load during fermentation could have an impact on the increased levels of yellowness after the fermentation stages. Fermentation, cooking, and the addition of sorghum malt at BSF had a positive impact on the yellowness of the beverage. The concertation of solids particles in Umqombothi does affect the yellowness, as observed after sieving, when the yellowness significantly (*p* < 0.05) increased for U1 and decrease for U2 and U3, respectively. The yellowness values are much higher than the redness values, which further suggests that Umqombothi is dominated by a yellow colour, as it is closer to a hue angle of 90 °C, which indicates pure yellowness on the colour scale.

**Table 3:** The effect of two-stage fermentation on yellowness values of Umqombothi.

Sampling stages	Yellowness		
	U1 (30-30 °C)	U2 (30-25 °C)	U3 (25–30 °C)
**Before first fermentation (BFF)**	4.27 ± 0.01^a^	4.77 ± 0.02^a^	5.00 ± 0.18^a^
**After first fermentation (AFF)**	5.12 ± 0.00^b^	5.76 ± 0.01^b^	4.77 ± 0.01^b^
**Before second fermentation (BSF)**	8.70 ± 0.01^c^	7.20 ± 0.02^c^	7.42 ± 0.01^c^
**After second fermentation (ASF)**	22.73 ± 0.02^d^	22.35 ± 0.00^d^	23.30 ± 0.01^d^
**The final product (FP)**	23.15 ± 0.04^e^	21.32 ± 0.00^e^	21.50 ± 0.00^e^
**Overall fermentation temperatures**	12.79 ± 8.71^a^	12.28 ± 8.12^b^	12.39 ± 8.53^c^

Mean value ± standard deviation of triplicate determination. Mean values in the same column followed by different letters are significantly (*p* ≤ 0.05) different.

The yellowness as affected by fermentation temperatures were 12.79, 12.28, and 12.39 for U1, U2 and U3 respectively and were significantly (p < 0.05) different. Umqombothi U1 had a significantly (*p* < 23.15) greater yellowness than U3 and U2, respectively. The constant high fermentation temperatures prove to have a positive impact on the colour of Umqombothi. According to Refs. [12,8] the colour of Umqombothi varies from pale buff to pink-brown to cream-colour after sieving, based on the naked eye.

The chroma attribute of Umqombothi from different fermentation temperatures, at different sampling stages is displayed in Table 4. The chroma of Umqombothi was positive during the production process for U1, U2 and U3. The chroma of sampling stages of BFF, AFF, BSF, ASF and FP ranged from 4.37 to 24.36, 4.89–23.65 and 4.87–24.63 for U1, U2 and U3, respectively. The chroma was significantly (*p* < 0.05) different during all sampling stages of the production process for U1, U2 and U3, respectively. The chroma as affected by fermentation temperatures were 13.43, 12.83, 12.93 and they were significantly (*p* < 0.05) different for U1, U2 and U3 respectively. U1 Umqombothi had a significantly (*p* < 13.43) greater chroma than U3 and U2 respectively.

**Table 4:** The effect of two-stage fermentation on chroma values of Umqombothi.

Sampling stage	Chroma		
	U1 (30-30 °C)	U2 (30-25 °C)	U3 (25–30 °C)
**Before first fermentation (BFF)**	4.37 ± 0.01^a^	4.89 ± 0.02^a^	5.10 ± 0.18^a^
**After first fermentation (AFF)**	5.24 ± 0.00^b^	5.88 ± 0.01^b^	4.87 ± 0.01^b^
**Before second fermentation (BSF)**	8.99 ± 0.01^c^	7.51 ± 0.04^c^	7.68 ± 0.01^c^
**After second fermentation (ASF)**	24.36 ± 0.02^d^	23.65 ± 0.00^d^	24.63 ± 0.00^d^
**The final product (FP)**	24.19 ± 0.05^e^	22.23 ± 0.00^e^	22.35 ± 0.00^e^
**Overall fermentation temperatures**	13.43 ± 9.31^a^	12.83 ± 8.59^b^	12.93 ± 9.02^c^

Mean value ± standard deviation of triplicate determination. Mean values in the same column followed by different letters are significantly (*p* ≤ 0.05) different.

The hue angle (h^o^) of Umqombothi from different fermentation temperatures, at different sampling stages is displayed in Table 5. The hue angle of Umqombothi was positive, during the production process of Umqombothi with U1, U2 and U3, respectively. The hue angle of sampling stages BFF, AFF, BSF, ASF and FP ranges from 68.96 to 78.11, 70.92–77.82 and 71.15–78.36 for U1, U2 and U3 respectively.

**Table 5:** The effect of two-stage fermentation on Hue angle (h^o^) values of Umqombothi.

Sampling stage	Hue		
	U1 (30-30 °C)	U2 (30-25 °C)	U3 (25–30 °C)
**Before first fermentation (BFF)**	77.74 ± 0.06^a^	77.35 ± 0.16^a^	78.36 ± 0.23^a^
**After first fermentation (AFF)**	78.11 ± 0.00^b^	77.82 ± 0.13^b^	78.36 ± 0.06^a^
**Before second fermentation (BSF)**	75.42 ± 0.05^c^	73.38 ± 0.33^c^	75.07 ± 0.06^b^
**After second fermentation (ASF)**	68.96 ± 0.09^d^	70.92 ± 0.00^d^	71.15 ± 0.00^c^
**The final product (FP)**	73.18 ± 0.01^e^	73.54 ± 0.00^c^	74.14 ± 0.00^d^
**Overall fermentation temperatures**	74.68 ± 3.48^a^	74.60 ± 2.71^a^	75.41 ± 2.83^b^

Mean value ± standard deviation of triplicate determination. Mean values in the same followed by different letters are significantly (*p* ≤ 0.05) different.

The hue angle as affected by fermentation temperatures were 74.68, 74.60 and 75.41 and they were significantly (*p* < 0.05) different for U1, U2 and U3, respectively. Umqombothi U3 had a significantly (*p* < 75.41) greater hue angle than U1 and U2. The increases in the microbial population at the end of the first fermentation and the removal of solid particles in the final product could cause increased the hue angle [22]. discovered a similar pattern in which temperature, polyphenol oxidation, and grist material all influenced the colour of the wort during the processing steps.

The colour differences (ΔE) of Umqombothi samples fermented at different temperatures ranged from 0.76 to 5.99. Colour difference (ΔE) < 1 is defined as a not noticeable difference, meaning that the observer does not detect the difference. A colour difference (ΔE = 1) is defined as a barely discernible change (JND).

The colour difference between 4 and 8 is perceptible but acceptable [23], which means that an observer notices the difference and considers it acceptable. The colour difference between Umqombothi samples at the different fermentation temperatures U1, U2 and U3, were perceivable with ΔE of 3.58, 2.07 and 2.02 for U1–U2, U1–U3 and U2–U3, respectively.

The pH obtained during the manufacturing process of Umqombothi is presented in Table 6. The pH obtained during all sampling stages showed a significant (*p <* 0.05) difference, with ASF and FP sampling stages for U3 not significantly (*p <* 0.05) different. However, for U2 a significant difference was only observed from BFF until ASF, thereafter, no further significant reduction was observed between ASF and FP. After cooking, a significant ((*p <* 0.05)) increase in pH for U1, U2 and U3 respectively was observed. Similar trends were observed by Ref. [8] after the cooking stage (BSF) during the fermentation of Umqombothi. After the second fermentation stage, a significant (*p <* 0.05) decrease in pH was observed for U1 and U3, while U2 showed a significant (*p <* 0.05) increase in pH within 24 h. Thereafter, a further significant (*p <* 0.05) decline in pH was observed for all three batches after the removal of the solid particles. The observed pH values reported in this study are similar to that reported by Ref. [8] for Umqombothi produced in homes, laboratories and township environments [2]. reported a similar pH of 3.67 in Umqombothi produced in Gauteng, South Africa. The findings also concur with those reported by Ref. [10].

**Table 6:** The effect of two-stage fermentation on pH values of Umqombothi.

Production stage	pH		
	U1 (30-30 °C)	U2 (30-25 °C)	U3 (25–30 °C)
**Before fermentation (BFF)**	5.99 ± 0.01^a^	6.00 ± 0.02^a^	6.02 ± 0.01^a^
**After fermentation (AFF)**	3.33 ± 0.02^b^	3.38 ± 0.02^b^	4.26 ± 0.01^b^
**Before second Fermentation (BSF)**	3.61 ± 0.01^c^	3.70 ± 0.01^c^	4.50 ± 0.00^c^
**After second fermentation (ASF)**	3.41 ± 0.01^d^	3.74 ± 0.01^d^	3.40 ± 0.01^d^
**The final product (FP)**	3.45 ± 0.00^e^	3.75 ± 0.00^d^	3.53 ± 0.01^e^
**Overall fermentation temperatures**	3.96 ± 1.06^a^	4.12 ± 0.98^b^	4.34 ± 0.97^c^

Mean value ± standard deviation of triplicate determination. Mean values in the same column followed by different letters are significantly (*p* ≤ 0.05) different.

When comparing the impact of pH as affected by fermentation temperatures there was a significant (*p <* 0.05) difference between U1, U2 and U3. The U1 Umqombothi batch produced a significantly (*p <* 3.96) lower pH compared to U2 and U3. A higher pH facilitates microbial growth which may affect the product's shelf life and organoleptic properties [24]. An increase in lactic acid bacteria during fermentation causes the reduction in pH in Umqombothi by the production of lactic acid and acetic acid [25,26]. The pH affects enzyme activity and is crucial in the liquefaction and conversion of malt starch into fermentable sugars [2].

Additionally, because higher pH encourages microbial growth, beverage with lower pH have a longer shelf life and higher quality than those with higher pH. Based on findings, it appears that U1 conditions are the most favourable for the fermentation of Umqombothi as it results in the lowest pH levels.

The total soluble solids (TSS) as affected by different fermentation temperatures during Umqombothi production are presented in Table 7. The TSS increased significantly (*p <* 0.05) from 1.00 to 1.10^o^Brix during the first fermentation for U1, U2 and U3. Before the second fermentation the TSS significantly (*p <* 0.05) increased to 8.00^o^Brix, 7.50°Brix and 7.00^o^Brix for U1, U2 and U3 respectively. This is due to the hydrolysis of starch into fermentable sugars during the cooking process, however, complete gelatinization of starch was not achieved. Starch in African beverages acts as a source of sugar, a thickener and a suspending agent [5]. Gelatinization and incomplete degradation of starch cause African beverages to be high in viscosity [5]. Umqombothi is cooked at 60 °C for 40 min, below the gelatinization of sorghum and maize starch which is between 67 and 81 °C and 63–67 °C, respectively [5]. For the enzymes to gain access to starch molecules, gelatinization must occur, where starch granules are broken down into fermentable sugars [27]. During the second fermentation, there was a significant increase (*p <* 0.05) of TSS from 8.00 to 10.00^o^Brix, 7.50–10.00^o^Brix and 7.00–10.00^o^Brix for U1, U2 and U3. Better access to the substrates (starch, protein and lipids), high water activity and surface area are the ideal conditions for enzymes to function optimally [28]. The TSS obtained in the final product was significantly (*p <* 0.05) lower for U1 and U2. However, there was no significant change in the TSS for U3.

**Table 7:** The effect of two-stage fermentation on total soluble solids values of Umqombothi.

Production stage	Total soluble solids (^o^Brix)		
	U1 (30-30 °C)	U2 (30-25 °C)	U3 (25–30 °C)
**Before first fermentation (BFF)**	1.00 ± 0.00^a^	1.00 ± 0.00^a^	1.00 ± 0.00^a^
**After first fermentation (AFF)**	1.10 ± 0.00^b^	1.10 ± 0.00^b^	1.10 ± 0.00^b^
**Before second fermentation (BSF)**	8.00 ± 0.00^c^	7.50 ± 0.00^c^	7.00 ± 0.00^c^
**After second fermentation (ASF)**	10.00 ± 0.00^a^	10.10 ± 0.10^d^	10.00 ± 0.00^d^
**The final product (FP)**	9.87 ± 0.12^d^	10.00 ± 0.00^a^	10.00 ± 0.00^a^
**Overall fermentation temperatures**	5.99 ± 4.24^a^	5.94 ± 4.24^b^	5.82 ± 4.18^c^

Mean value ± standard deviation of triplicate determination. Mean values in the same column followed by different letters are significantly (*p* ≤ 0.05) different.

A similar pattern in the decrease in the TSS was reported by Ref. [26], by the removal of the second conversion malt [18]. reported a decrease in TSS from 9.7^o^Brix to 8.0^o^Brix on the sample stored between 28 °C and 30 °C. According to Ref. [18] the TSS act as nutrients used for bacterial growth, as the bacterial numbers increase, the TSS decrease.

When comparing the impact of TSS as affected by fermentation temperatures there was a significant (*p* < 0.05) different for U1, U2 and U3 respectively. The U2 and U3 Umqombothi batches had a significantly (*p <* 10.00) higher TSS value than U1. However, U1 showed a significant (*p* < 0.05) decrease in TSS as seen by the levelling of solids in the products at the FP sampling stage, not all dissolved solids were available for use by the microorganisms present. It's possible that the levelling out was due to the inhibitory effect of ethanol. Yeast and LAB use fermentable sugars as a source of food to create ethanol and carbon dioxide [29]. This is like Tchapalo (Ivorian sorghum beer), which shows a significant (*p <* 0.05) decrease in TSS at ambient temperature (28–30 °C) after the first 24 h from 9.7^o^Brix to 8.0^o^Brix [18]. There was a significant difference (*p <* 0.05) at the TSS of Umqombothi as affected by fermentation temperatures. Sample U1 had a significantly (*p <* 5.99) higher TSS than U3 and U2 respectively. Cooking the soured porridge for a suitable period is required for starch gelatinization and the release of nutrients bound up in yeast cells [16]. The cooking duration was discovered to influence the alcohol concentration, TSS, and pH. The breakdown of cooked starch to fermentable sugars by endogenous amylolytic enzymes drives the development of fermentative microorganisms. The quantity of TSS increases as the endosperm protein surrounding the starch granules softens (during gelatinization), the grain was moved to the retting water [16]. This might explain the rising trend in TSS levels with greater cooking and fermentation time. Total soluble solids will be increased systemically throughout the production stages, and Umqombothi production will be improved with U1 fermentation temperatures.

The ethanol content and specific gravity of different fermentation temperatures at different sampling stages of the production process are presented in Table 8. Beer authenticity can be identified by percentage alcohol content by volume (% ABV) and original gravity, as they are highly indicative parameters. Specific gravity (SG) is also a tool to measure the fermentation process [30]. The higher the SG, the better the sugar will dissolve in the liquid (wort). The Specific gravity for all sampling stages resulted in no significant difference for batches U1, U2 and U3.

**Table 8:** The effect of two-stage fermentation on specific gravity (SG) values of Umqombothi.

Production stage	Specific gravity		
	U1 (30-30 °C)	U2 (30-25 °C)	U3 (25–30 °C)
**Before first fermentation (BFF)**	1.00 ± 0.00^a^	1.00 ± 0.00^a^	1.00 ± 0.00^a^
**After first fermentation (AFF)**	1.00 ± 0.00^a^	1.01 ± 0.00^a^	1.01 ± 0.00^a^
**Before second fermentation (BSF)**	1.04 ± 0.00^b^	1.03 ± 0.00^a^	1.03 ± 0.00^a^
**After second fermentation (ASF)**	1.04 ± 0.00^b^	1.04 ± 0.00^a^	1.04 ± 0.00^a^
**The final product (FP)**	1.04 ± 0.00^b^	1.04 ± 0.00^a^	1.04 ± 0.00^a^
**Overall fermentation temperatures**	1.02 ± 0.02^a^	1.02 ± 0.02^a^	1.02 ± 0.02^a^

Mean value ± standard deviation of triplicate determination. Mean values in the same column followed by different letters are significantly (*p* ≤ 0.05) different.

The original gravity (OG) minus the final gravity (FG) of the wort can approximately provide the percentage alcohol content by volume (% ABV) of the final beverage. Specific gravity can be used to determine the alcohol % of the beer [31,32].

An ethanol content of 2.00 % was obtained for all final product samples. The alcohol content (%) reported in this study agrees with the 2–3.5 % reported by Ref. [10] but is lower (2.6 %) when compared to that reported by Ref. [8]. According to Ref. [10], the ethanol content of Umqombothi will differ depending on where and how it was brewed [12]. reported alcohol content of 1–8%, whereas values of 2.5–4.5 % are the most common. Ethanol characteristics contribute to flavour and impacts the quality of the beer [33]. [32] mentioned that microbial activity of conversion of ethanol to acetic acid can cause a decrease in ethanol content as the beer deteriorates. A greater fermentation temperature, in general, impacts the pace of sugar metabolism (i.e., results in a quick increase in alcohol content and other by-products like volatile compounds) [16]. In this study, however, a greater fermentation temperature combination, U1 resulted in the same alcohol concentration when compared to U2 and U3.

#### 2.1.2 Microbiological population in umqombothi

The LAB counts during Umqombothi production for different fermentation temperatures are displayed in Table 9. The lactic acid bacteria numbers significantly (*p <* 0.05) increased during the first fermentation for U1, U2 and U3 respectively [10]. reported lactic acid bacteria are the most dominant microorganisms during the fermentation of Umqombothi, with fewer occurrences and reports of yeast and fungi which is noticeable in this study.

**Table 9:** The effect of two-stage fermentation on lactic acid bacteria (LAB) values of Umqombothi.

sampling stage	LAB (cfu/ml)		
	U1 (30-30 °C)	U2 (30-25 °C)	U3 (25–30 °C)
**Before first fermentation (BFF)**	2.50 ± 0.05^a^	2.30 ± 0.17^a^	2.50 ± 0.04^a^
**After First fermentation (AFF)**	7.22 ± 0.03^b^	7.79 ± 0.03^b^	8.10 ± 0.05^b^
**Before second fermentation (BSF)**	0.00 ± 0.00^c^	0.00 ± 0.00^c^	0.00 ± 0.00^c^
**After second fermentation (ASF)**	7.94 ± 0.05^d^	8.12 ± 0.05^d^	8.12 ± 0.05^d^
**The final product (FP)**	8.24 ± 0.06^e^	8.06 ± 0.02^d^	8.06 ± 0.05^b^
**Overall fermentation temperatures**	5.18 ± 3.44^a^	5.25 ± 3.55^b^	5.36 ± 3.57^c^

Mean value ± standard deviation of triplicate determination. Mean values in the same column followed by different letters are significantly (*p* ≤ 0.05) different.

The environment in which LAB thrives is rich in proteins, sugars, vitamins, nucleic acid, and lipids typically found in sorghum beverages which therefore explain their predominance in the sorghum microflora [10]. The growth rate can also be linked to relative superiority in starchy sorghum substrate utilization, as well as veritable carbohydrate metabolisms as well as strong acid tolerance. Lactic acid bacteria significantly (*p <* 0.00) decreased or were non-detected at the BSF stage for U1, U2 and U3, respectively, which may be to cooking (BSF sampling stage).

Cooking (Before second fermentation) involves the conversion of starch into simple sugars that can be metabolized by LAB into alcohol and carbon dioxide and could result in an increase in LAB counts [34]. The overall fermentation values of LABs were significantly (*p* < 0.05) different for U1, U2 and U3 respectively.

The U3 sample had significantly (*p <* 5.36) higher LAB value than U2 and U3 respectively. During cooking (BSF), starch is converted to fermentable sugars, amino acids, and vitamins, which aids in the development of LAB and yeast to produce flavour, and fragrance, and preserve the Umqombothi beverage sensory quality profile [2,35]. The addition of sorghum malt and fermented beer powder as inoculum, before the second fermentation stage (BSF), increased the product's total flora. Previously, LAB in the production of opaque sorghum beverages showed the same trend [35]. This category of microorganisms, according to Ref. [10], are exploitative competitors that impede other microorganisms by rapidly utilizing glucose and accumulating acetic and lactic acid. Lactic acid bacteria are one of the most common microorganisms used in sorghum fermentation, with LAB being the primary carrier. As the fermentation temperature was reduced to 25 °C for the first fermentation, the LAB count for the final product was significantly (*p <* 0.05) decreased to 8.06 log cfu/ml. As noted in a study by Refs. [2,8], LAB and yeast are the dominant microorganisms in Umqombothi, with the highest LAB counts present at the lowest pH. In the current study, U1 exhibited a LAB count of 8.24 log cfu/ml in the final product at the lowest pH of 3.96 than U2 and U3.

The TVC counts during Umqombothi production for different fermentation temperatures are displayed in Table 10. The TVC of sampling stages of BFF, AFF, BSF, ASF and FP ranged from 0.00 to 8.46 log cfu/ml, 0.00–8.19 log cfu/ml and 0.00–8.18 log cfu/ml for U1, U2 and U3, respectively. The TVCs significantly (*p <* 0.05) increased after the fermentation stages for U1, U2 and U3, respectively. The TVCs significantly (*p <* 0.00) reduced to non-detected at BSF sampling stages for all three fermentation temperatures. The significant (*p <* 0.05) decrease in TVC in the final product for U1, U2 and U3, could be caused by sieving.

**Table 10:** The effect of two-stage fermentation on total viable count (TVCs) values of Umqombothi.

Sampling stage	Total viable counts (cfu/ml)		
	U1 (30-30 °C)	U2 (30-25 °C)	U3 (25–30 °C)
**Before first fermentation (BFF)**	4.63 ± 0.06^a^	4.83 ± 0.04^a^	4.85 ± 0.02^a^
**After first fermentation (AFF)**	7.10 ± 0.06^b^	7.09 ± 0.02^b^	8.18 ± 0.03^b^
**Before second fermentation (BSF)**	0.00 ± 0.00^c^	0.00 ± 0.00^c^	0.00 ± 0.00^c^
**After second fermentation (ASF)**	8.18 ± 0.01^d^	8.18 ± 0.07^d^	8.17 ± 0.14^b^
**The final product (FP)**	8.46 ± 0.01^e^	8.19 ± 0.03^d^	8.09 ± 0.39^b^
**Overall fermentation temperatures**	5.68 ± 3.25^a^	5.66 ± 3.19^a^	5.86 ± 3.31^b^

Mean value ± standard deviation of triplicate determination. Mean values in the same column followed by different letters are significantly (*p* ≤ 0.05) different.

A combination of cooking and fermentation, as performed in the case of Umqombothi, improves the nutrient quality of sorghum and reduces the content of anti-nutritional components to a safe level [10,36]. The high moisture content, nutrients, and contamination with microorganisms from raw material contribute to the significant increase in TVC in opaque sorghum traditional beverages. The inclusion of tainted sorghum malt after heating would have likely resulted in a huge increase in the TVC in the BSF and FP sampling stages. The observed TVC counts reported in this study are lower than the 10.8 logs cfu/ml reported by Ref. [34].

TVC is commonly used as an indicator to evaluate the quality, safety, and shelf-life of food [37]. The relevant microorganisms can grow and survive under oxygenated environments and are considered mesophilic bacteria, as they have a wide growth temperature range of 20–45 °C but mainly grow optimally at 35–37 °C [29]. Excessive growth of total aerobes (spoilage bacteria) may lead to spoilage of Umqombothi and restriction of its shelf life, resulting in undesirable sensory characteristics, such as loss of texture, off-flavours and colour [2,37]. Total plate count colonies, on the other hand, indicate the collective enumeration of all mesophilic bacteria, including lactic acid bacteria, yeast, and mould, as well as total coliforms.

The yeast counts during Umqombothi production for different fermentation temperatures are displayed in Table 11. Brewing yeast has a direct impact on the character and quality of beer, depending on which type is used to make a particular style. Traditional fermented beverages could be a valuable source of yeast for the brewing business. In addition to *S. cerevisiae*, many traditional fermented beverages and other beverages start spontaneously and frequently contain a mix of wild yeast strains. Yeast is responsible for both the conversion of fermentable carbohydrates into ethanol and the formation of a large variety of flavour-active chemicals [38]**.**

**Table 11:** The effect of two-stage fermentation on yeast values of Umqombothi.

Sampling stage	Yeast (cfu/ml)		
	U1 (30-30 °C)	U2 (30-25 °C)	U3 (25–30 °C)
**Before first fermentation (BFF)**	3.49 ± 0.03^a^	3.64 ± 0.08^ae^	3.67 ± 0.03^a^
**After first fermentation (AFF)**	7.56 ± 0.05^b^	7.54 ± 0.05^b^	7.51 ± 0.01^b^
**Before second fermentation (BSF)**	0.00 ± 0.00^c^	0.00 ± 0.00^c^	0.00 ± 0.00^c^
**After second fermentation (ASF)**	3.68 ± 0.07^d^	3.28 ± 0.01^de^	5.37 ± 0.07^d^
**Final product (FP)**	3.41 ± 0.04^e^	3.53 ± 0.29^eda^	5.75 ± 0.07^e^
**Overall Fermentation temperature**	3.63 ± 2.48^a^	3.60 ± 2.48^b^	4.46 ± 2.63^c^

Mean value ± standard deviation of triplicate determination. Mean values in the same column followed by different letters are significantly (*p* ≤ 0.05) different.

The yeast counts of sampling stages of BFF, AFF, BSF, ASF and FP ranged from 0.00 to 7.56 log cfu/ml, 0.00–7.54 log cfu/ml and 0.00–7.51 log cfu/ml for U1, U2 and U3, respectively. The yeast counts of the respective sampling stages differed significantly (*p <* 0.05) for U1 and U3. The significant (*p <* 0.05) decrease of yeast counts for U1 and significant (*p <* 0.05) increase for U2 and U3, in the FP sampling stages could be caused by sieving (removal of solid particle sizes), this confirmed the fact that most microorganisms are from the ingredients, solids. Umqombothi is nutrient-rich, which explains why yeast numbers increase dramatically after fermentation [10,12,8]. Fermentation normally causes enzyme activation, a decrease in pH, and an increase in metabolic and microbial activity.

This causes the substrate to break down, boosting the nutritional quality of the beverage, which favours yeast growth [10]. The yeast count in relation to the overall fermentation temperatures were significantly (*p <* 0.05) different. Sample U3 had a significantly (*p <* 4.46) higher yeast count than U1 and U2 respectively.

The multiplication of yeasts is fuelled by endogenous amylolysis, which converts cooked starch to fermentable sugars. The fermentation period has a large impact on the quality of the product. Due to the presence of LAB, the lower the alcohol concentration, pH, and viscosity, the longer the fermentation was permitted to continue [16]. The observed yeast counts reported in this study are lower in comparison to the 6.52 log cfu/ml, 7.1 log cfu/ml, and 6.42 log cfu/ml reported by Ref. [8]. Which may be due to the fermentation time. In this study, the fermentation period was 24 h as compared to the 48 h used by Ref. [8]. Yeast and moulds form part of a large group of fungi and can produce mycotoxins, which pose a health hazard to humans. Effect of mycotoxins range from acute poisoning to long-term effects such as immune deficiency and cancer [2]. According to Ref. [2], *S. cerevisiae* is one of the most dominant organisms in the spontaneous fermentation of Umqombothi. Yeast counts are higher than most other microorganisms, except for LAB and TVC. The strains of this species influence the flavour and aroma characteristic of Umqombothi [39]. Yeast thrives in high water activity, and warm acidic environments (20–30 °C; pH 4.5–6.5), while moulds grow between pH 2–8.5 [40,39]. Yeast is the only living microorganism that can change from respiratory to fermentation metabolism. If glucose is present, it will always take a fermentative route to utilize it, despite the availability of oxygen [41]. *Saccharomyces cerevisiae* traditionally conducts top fermentations where yeasts congregate on the surface of the fermenting wort [39]. These growth conditions of yeast and moulds render Umqombothi a selective environment for their growth. Umqombothi exhibited yeast and mould counts of 3.41 log cfu/ml, 3.52 log cfu/ml, 5.75 log cfu/ml and 2.14 log cfu/ml, 2.61 log cfu/ml, 0.00 log cful/ml for yeast and mould at U1, U2 and U3 fermentation temperatures, respectively. The microbial counts in the final product result in Umqombothi being susceptible to rapid deterioration, and a possible explanation for the short shelf life of 2–3 days [42,43].

Traditional fermented beverages could be a valuable supply of yeast for the brewing industry. The role of a yeast strain in product quality is frequently underestimated, yet it has a significant impact on beverage character as a raw material [38]. Most microorganisms in Umqombothi are derived from raw materials, such as sieving (removal of solid particles, which leave the supernatant and include the majority of the microorganisms, have an impact on the chemical analysis and microbial counts.). The relationship between yeast count and pH in this study, show that the lower the pH the lower the yeast counts, in the U1 yeast counts 3.41 log cfu/ml, 3.52 log cfu/ml and 5.75 log cfu/ml, pH 3.96, 4.12 and 4.34 for U1, U2 and U3.

The mould counts during Umqombothi production for different fermentation temperatures are displayed in Table 12**.** The mould counts of sampling stages BFF, AFF, BSF, ASF and FP ranged from 0.00 to 6.81 log cfu/ml, 0.00–6.73 log cfu/ml and 0.00–6.77 log cfu/ml for U1, U2 and U3, respectively. The mould counts of the respective sampling stages differed significantly (*p <* 0.05) for U1 and U2. The significant (*p <* 0.00) reduction of the microbial count to a point that it is non-detectable at BSF for U1, U2 and U3, could be due to cooking time and temperature. The purpose of cooking is to reduce the number of bacteria [27]. The decrease in nutrients, due to microorganism competition of food and lower pH of the final product and the removal of solid particles affected mould counts and a significant (*p <* 0.05) increase for U2.

**Table 12:** The effect of two-stage fermentation on mould values of Umqombothi.

Sampling stage	Mould (cfu/ml)		
	U1 (30-30 °C)	U2 (30-25 °C)	U3 (25–30 °C)
**Before first fermentation (BFF)**	3.04 ± 0.04^a^	2.91 ± 0.02^a^	3.09 ± 0.08^a^
**After first fermentation (AFF)**	6.81 ± 0.13^b^	6.73 ± 0.15^b^	6.77 ± 0.28^b^
**Before second fermentation (BSF)**	0.00 ± 0.00^c^	0.00 ± 0.00^c^	0.00 ± 0.00^c^
**After second fermentation (ASF)**	2.88 ± 0.03^d^	1.73 ± 0.05^d^	3.60 ± 0.52^d^
**The final product (FP)**	2.14 ± 0.12^e^	2.62 ± 0.15^e^	0.00 ± 0.00^c^
**Overall fermentation temperatures**	2.98 ± 2.28^a^	2.79 ± 2.29^b^	2.69 ± 2.63^b^

Mean value ± standard deviation of triplicate determination. Mean values in the same column followed by different letters are significantly (*p* ≤ 0.05) different.

The overall fermentation temperatures values for mould count were significantly (*p <* 0.05) different. The U1 exhibited a significantly (*p <* 2.98) higher mould count than U2 and U3, respectively. Umqombothi is susceptible to rapid deterioration due to mould growth, which could be one of the reasons Umqombothi has a shelf life of 2–3 days [42,43].

The total coliform count during Umqombothi production for different fermentation temperatures is displayed in Table 13. Coliforms that occur in large numbers guide the presence of potential entero-pathogens (*E. coli*) in drinking water, soil and vegetables, indicative of their microbiological quality [37,44]. Coliforms can be described as aerobic and facultative anaerobic bacteria, and for these organisms to multiply, they need to ferment lactose under gassy and acidic conditions in a temperature range of 35–37 °C [24,37]. The total coliforms of sampling stages of BFF, AFF, BSF, ASF and FP range from 0.00 to 4.41 log cfu/ml, 0.00–4.26 log cfu/ml, and 0.00–7.73 log cfu/ml for U1, U2 and U3 respectively.

**Table 13:** The effect of two-stage fermentation on total coliform values of Umqombothi.

Sampling stage	Total coliforms (cfu/ml)		
	U1 (30-30 °C)	U2 (30-25 °C)	U3 (25–30 °C)
**Before first fermentation (BFF)**	3.39 ± 0.14^a^	4.26 ± 0.05^a^	3.78 ± 0.04^a^
**After first fermentation (AFF)**	4.41 ± 0.06^b^	3.80 ± 0.57^b^	7.73 ± 0.08b
**Before second fermentation (BSF)**	0.00 ± 0.00^c^	0.00 ± 0.00^c^	0.00 ± 0.00c
**After second fermentation (ASF)**	0.00 ± 0.00^c^	0.00 ± 0.00^c^	0.00 ± 0.00^c^
**The final product (FP)**	0.00 ± 0.00^c^	0.00 ± 0.00^c^	0.00 ± 0.00^c^
**Overall fermentation temperatures**	1.51 ± 1.93^a^	1.61 ± 2.06^a^	2.30 ± 3.19^b^

Mean value ± standard deviation of triplicate determination. Mean values in the same column followed by different letters are significantly (*p* ≤ 0.05) different.

Consequently, the evaluation of total coliforms in Umqombothi was necessary, as water was one of the main ingredients in the production of Umqombothi [45]. Total coliforms were significantly (*p <* 0.00) non-detected at BSF, ASF and FP sampling stages for U1, U2 and U3 respectively. These findings demonstrated that certain stages of heating and fermenting have a critical role in the reduction of coliform bacteria during the production process [35].

The overall fermentation temperatures U1, U2 and U3, and they were significantly (*p <* 0.05) different. U3 exhibited a significantly (*p <* 2.30) higher total coliform count than U2 and U1, respectively. The higher fermentation temperature in the Umqombothi production process did not favour the coliform growth. This condition is the reason for the significant (*p <* 0.05) decrease in coliforms at U1.

#### 2.1.3 Sensory characteristics of umqombothi

**Fig. 2:**
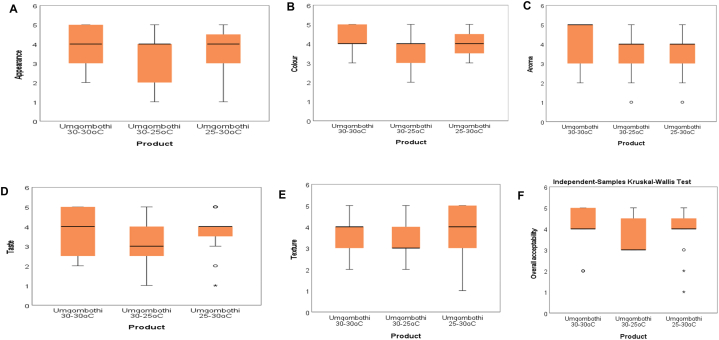
Kruskal Willis test for sensory attributes, a scale of 1–5 (1- Dislike very much, 2-Dislike moderately, 3-Neither like or Dislike, 4-Like moderately and 5-Like very much).

The results of the sensory evaluation of Umqombothi as affected by the two-stage fermentation process in three different fermentation temperatures are presented in Fig. 2 and Table 14. The Umqombothi appearance distributions (Fig. 2A) (30-30 °C), (30-25 °C), and (25–30 °C) are symmetric, and the median of consumer panellists is the same, 4-Like moderately. The interquartile range for (30-30 °C) and (30-25 °C) is approximately 2, while the interquartile range for (25–30 °C) is approximately 1.5. The range for (30-30 °C) is 3, while the range for (30-25 °C) and (25–30 °C) is 4. In terms of appearance, all three products had the same median (average). According to the appearance data, (30-25 °C) and (25–30 °C) are more spread, with a larger spread of the middle 50 % for (30-30 °C) and (30-25 °C). Overall, the appearance ranged from (30-25 °C).

**Table 14:** Umqombothi Kruskal ranks.

Sensory attributes	Umqombothi		
	U1 (30-30 °C)	U2 (30-25 °C)	U3 (25–30 °C)
**Appearance**	25.43^a^	19.10^a^	24.47^a^
**Colour**	28.20^a^	19.00^a^	21.80^a^
**Aroma**	26.90^a^	21.50^a^	20.60^a^
**Taste**	25.77^a^	18.63^a^	24.60^a^
**Texture**	24.10^a^	18.70^a^	26.20^a^
**Overall acceptability**	25.93^a^	19.97^a^	23.10^a^

Mean value ± standard deviation of triplicate determination. Mean values in the same row followed by different letters are significantly (*p* ≤ 0.05) different. Sensory distribution for attributes.

The median number of consumer panellists for colour (Fig. 2B) is the same for all three Umqombothi samples, 4-Like moderately. The interquartile for all three Umqombothi samples was one, and the ranges for (30-30 °C) and (25–30 °C) were two, while the range for (30-25 °C) was three. According to the colour data, the spread of the middle 50 % was the same for all three samples, with (30-25 °C) being the most spread. The (30-30 °C) sample had a higher median, resulting in a better aroma average (Fig. 2C) of 5-Like very much than the (30-25 °C) and (25–30 °C) samples of 4-Like moderately. Based on the total data, the sample aromas are evenly distributed, with the middle 50 % having a larger spread (30-30 °C). The (30-30 °C) sample has a higher median, resulting in a better taste average (Fig. 2D) of 4-like moderately than the (25–30 °C) and (30-25 °C) samples, which have 3-Neither like nor dislike. According to the data, (30-25 °C) taste is more widely distributed than (30-30 °C) and (25–30 °C), respectively. The middle 50 % spread is wider (30-30 °C). For the most part, the values are on the upper end of this range (25–30 °C). The upper and lower outliers were observed in (25–30 °C).

Samples (30-30 °C) and (25–30 °C) have a texture median (average) of (Fig. 2E) 4-Like moderately compared to (30-25 °C) of 3-Neither like nor dislike. According to the data, (25–30 °C) has the largest range, thus more spread and the spread of the middle 50 % is larger.

The samples (30-30 °C) and (25–30 °C) have a higher overall acceptability median (average) of (Fig. 2F) 4-Like moderately than the sample (30-25 °C) of 3-Neither like nor dislike. According to the data (30-25 °C), overall acceptability has a wider range and thus is more spread. Because the sample (30-25 °C) has an interquartile of 1.5, the spread of the middle 50 % is greater.

## 3 Conclusion

Umqombothi was successfully produced from three different fermentation temperatures, using a two-stage fermentation process. Sample U1(30-30 °C), appears to be the preliminary correct approach to ferment Umqombothi, with the lowest pH which suppresses the growth of spoilage microorganisms and exhibits positive effect on the shelf life of the fermented beverages. Additionally, it was shown that the addition of starter culture and red sorghum malt helped to reintroduce microorganisms, demonstrating the effectiveness of the cooking step in lowering the microbial load. Additionally, it is found that the amount of solids in the beverage affects both the microbiological load and stability (syneresis), making the sieving stage (pore sizes) extremely important to the stability and shelf life of the beverage. Furthermore, as the results matched those of other authors who examined traditional umqombothi from the townships, it has been determined that, regardless of the location of production and the sanitary practises followed, the microbiota's development pattern is essentially the same. Therefore, Umqombothi in this current study is light, yellow, and red to creamy colour after sieving as compared to visually observation of pale buff, pink-brown to cream after sieving reported in other studies. However, complementary studies on Umqombothi beverage conservation are needed. Improved control of fermentations and product characteristics is strongly advised to preserve and sustain South African indigenous fermented beverages. This includes adding all ingredients prior to the cooking stage, sieving or "filtering" residue prior to the second fermentation stage, and using purified starter cultures with the right technological properties. As a result, conditioning or force carbonation and pasteurization would be simpler, making the beverage more stable and offering a longer shelf life.

### Data availability statement

Data generated in this study is the intellectual property of CPUT and is deposited in the CPUT library repository. The data will be available upon request.

### Complete ethics statement

This study was reviewed and approved by [Cape Peninsula University of Technology, Faculty of Applied Science Research Committee], with the approval number: [205221289/04/2022].

All participants/patients (or their proxies/legal guardians) provided informed consent to participate in the study.

All participants/patients (or their proxies/legal guardians) provided informed consent for the publication of their anonymised case details and images.

## CRediT authorship contribution statement

**Thembelani Xolo:** Writing - review & editing, Writing - original draft, Methodology, Investigation, Formal analysis, Data curation. **Zanephyn Keyser:** Writing - review & editing, Supervision, Methodology, Data curation, Conceptualization. **Victoria A Jideani:** Writing - review & editing, Supervision, Methodology, Data curation, Conceptualization.

## Declaration of competing interest

The authors declare that they have no known competing financial interests or personal relationships that could have appeared to influence the work reported in this paper.
