# Supplementary material for: Physicochemical and microbiological changes during two-stage fermentation production of umqombothi
Source: Heliyon. 2024 Jan 13;10(2):e24522. doi: 10.1016/j.heliyon.2024.e24522 (PMC10803943; doi:10.1016/j.heliyon.2024.e24522)
Supplement: Multimedia component 1 [file mmc1.pdf]

Statement of Permission

A site permit is required for this study.

**Reference no.**	205221289/04/2022
**Surname & name**	Xolo, Thembelani
**Staff Number**	205221289
**Program**	Master of Technology: Food and Science and Technology
**Title**	Effect of particle size, lactic acid bacteria and yeast strain on stability and sensory characteristic of umqombothi
**Supervisor(s)**	Dr. Z. Keyser
**FRC Signature**	
**Date**	April 06, 2022

Cape Peninsula University of Technology

P.O. Box 1906 · Bellville 7535 South Africa ·Tel: +27 21 953 8677 (Bellville), +27 21 460 4213 (Cape Town)

Conditional Ethics Approval Letter **Reference no: 205221289/04/2022**

**Office of the Chairperson**<br><br>**Research Ethics Committee**	**Faculty of Applied Sciences**

On 06 April 2022, the Faculty Research Ethics Committee of the Faculty of Applied Sciences has granted conditional ethics approval to Xolo, Thembelani for research activities related to a project to be undertaken for a degree (Master of Technology: Food and Science and Technology) at the Cape Peninsula University of Technology.

**Title of project:**	Effect of particle size, lactic acid bacteria and yeast strain on stability and sensory characteristic of umqombothi

The conditions for this ethics approval are:

1. The researchers must adhere to the IFST Guidelines for Ethical and Professional Practices for the Sensory Analysis of Foods.
2. To reduce risks to participants, test samples and products should be assessed for potential microbial, chemical, and physical hazards and certified safe before conducting the sensory evaluation.
3. This permission is granted for the duration of the study.
4. Research activities are restricted to those detailed in the research proposal.
5. The research team must comply with conditions outlined in AppSci/ASFREC/2015/1.1 v1, CODE OF ETHICS, ETHICAL VALUES AND GUIDELINES FOR RESEARCHERS.

[signature]	**06/04/2022**
**Signed: Chairperson: Research Ethics Committee**	**Date**

Department of Food Science and
Technology
P. O. Box 1906
Bellville 7535

Cape Peninsula University of Technology

INFORMED CONSENT FOR UMQOMBOTHI RESEARCH

Hello

We are scientists from Cape Peninsula University of Technology. We are conducting a research to standardizing the production of South African traditional fermented alcoholic beverage “Umqombothi”. No value will be added to any produce except the consumers endorse it. Hence, we are approaching you to be part of this study. We realize you need to make an informed decision whether or not to be part of this study, hence we have provided below further details with regards to the research to assist in your decision process.

Title of Research Project:
EFFECT OF PARTICLE SIZE, LACTIC ACID BACTERIA AND YEAST STRAIN ON STABILITY AND SENSORY CHARACTERISTIC OF UMQOMBOTHI

Investigator:

Thembelani Xolo	Tel:	0838749463	email:xolot@cput.ac.za
Dr. Zanephyn Keyser	Tel:	021 9538636	email:keyserz@cput.ac.za

Purpose of the Research:

There are many different types of beer (clear lager) that are produced and consumed around the world. Most of them are different from other different beers that are consumed in different parts of Africa. Malted sorghum, millet or maize are used to make African traditional beers and they are much thicker than clear beer with a distinctive sour yoghurt like refreshing taste. Large amount of solid particle causes these beers to opaque and pink-brown in colour. Alcohol content of indigenous beers normally vary between 2 and 3.5 % and characterized by short shelf life 2-3 days and consumed in its active state of fermentation on daily basis. The aim of the study is to determine the impact of fermentation time and temperature, ingredient particle size, lactic acid bacteria and Saccharomyces cerevisiae on the stability and sensory characteristics of Umqombothi.

Description of the Research:
This is an invitation to participate in the sensory study. The procedure to be adopted in the study as well as the terminologies on the score form will be explained to the panellists prior to tasting sessions. You will receive two traditional beer samples which were produced with the same ingredient but fermented at different temperature. You will be required to taste them and rate your preference (on a simple questionnaire) for each based on appearance, colour, taste, aroma, texture and overall acceptability. Each tasting session will last for 15-30 minutes depending on individual. The evaluation test will take place in the sensory evaluation laboratory at food technology building, in Bellville campus.

Potential Harm, Injuries, Discomforts, or Inconvenience:
Umqombothi is the tradition alcoholic beverage, excessive alcohol use can impairs driving ability and lead to the development of chronic diseases and other serious problems including:

- High blood pressure, heart disease, stroke, liver disease, and digestive problems.
- Cancer of the breast, mouth, throat, voice box, liver, colon, and rectum.
- Weakening of the immune system, learning and memory problems, including poor school performance.

Umqombothi is a traditional fermented alcoholic beverage and its popular among black people in South Africa. It is cream coloured after sieving, and opaque with yoghurt like flavour. Therefore, there is no known harm associated with tasting Umqombothi products in this study. However, even though it has not been proven to be allergenic it is suggested that people who are allergic to alcohol and corn should refrain from tasting.

Potential Benefits:
You will not benefit directly from participating in this study.

Confidentiality:
Confidentiality will be respected and no information that discloses the identity of the participant will be released or published.

Participation:
Participation in this research is voluntary. If you choose to participate in this study you may withdraw at any time.

Contact
If you have any questions about this study, please contact:

Thembelani Xolo	Tel:	0838749463	email:xolot@cput.ac.za
Dr. Zanephyn Keyser (super visor)	Tel:	021 9538636	email:keyserz@cput.ac.za

Consent:
By signing this form, I agree that:

1. The study was explained to me and all my questions answered.
2. I have the right to participate and the right to stop at any time.
3. I have been told that my personal information will be kept confidential
4. There is no likely harm from tasting beer to which kelp had been added.

I hereby consent to participate in this study:

....	....
**Name of Participant**	**Signature & Date**
....	....
**Name of Researcher**	**Signature & Date**

UMQOMBOTHI PRODUCT EVALUATION FORM

Instruction: **You are provided with 3 samples of South African traditional alcoholic fermented beverage “Umqombothi”. Please take a sip of water before you start tasting and in between tasting the different samples. Please rate each sample on its own merit based on the given attributes. Do not compare the samples**

Name of product: South African traditional fermented beverage “Umqombothi” ……………. Code:

	**Dislike very much (1)**	**Dislike moderately (2)**	**Neither Like nor Dislike (3)**	**Like moderately (4)**	**Like very much (5)**
Appearance					
Colour					
Aroma					
Taste					
Texture					
Overall acceptability					

Comments:

……

……

Name of product: South African traditional fermented beverage “Umqombothi”.......……… Code:

	**Dislike very much (1)**	**Dislike moderately (2)**	**Neither Like nor Dislike (3)**	**Like moderately (4)**	**Like very much (5)**
Appearance					
Colour					
Aroma					
Taste					
Texture					
Overall acceptability					

Comments:

……

……

Name of product: South African traditional fermented beverage "Umqombothi" ......……….... **Code**:

	**Dislike very much (1)**	**Dislike moderately (2)**	**Neither Like nor Dislike (3)**	**Like moderately (4)**	**Like very much (5)**
Appearance					
Colour					
Aroma					
Taste					
Texture					
Overall acceptability					

Comments:

....

....

We would like to obtain information about you. Kindly complete this brief questionnaire appropriately:

1. Do you have any allergic reaction to alcohol and or corn? Yes ☐ No ☐
   If yes, please refrain from the tasting

2. What is your gender? Female ☐ Male ☐

3. What is your race? Black ☐ Coloured ☐ White ☐ Indian ☐ Other ☐

4. Are you a student or staff? Student ☐ Staff ☐

5. If you are a student, are you an international student? Yes ☐ No ☐

6. What is your age group?

☐ Less than 20-29
☐ 30-39
☐ 40 & above

Thank you for assisting us!!!!
